# Rethinking morbidity compression

**DOI:** 10.1007/s10654-020-00642-3

**Published:** 2020-05-16

**Authors:** Rosie Seaman, Andreas Höhn, Rune Lindahl-Jacobsen, Pekka Martikainen, Alyson van Raalte, Kaare Christensen

**Affiliations:** 1 Max Planck Institute for Demographic Research, Konrad-Zuse Str. 1, Rostock, Germany; 2 Faculty of Social Sciences, University of Stirling, Stirling, UK; 3 Institute of Genetics and Molecular Medicine, University of Edinburgh, Edinburgh, UK; 4 Department of Epidemiology, Biostatistics, and Biodemography, University of Southern Denmark, Odense, Denmark; 5 Interdisciplinary Centre On Population Dynamics, University of Southern Denmark, Odense, Denmark; 6 Population Research Unit, University of Helsinki, Helsinki, Finland; 7 Danish Aging Research Centre, University of Southern Denmark, Odense, Denmark

**Keywords:** Morbidity compression, Ageing and health, Age at morbidity onset, Hospital admission

## Abstract

Studies of morbidity compression routinely report the average number of years spent in an unhealthy state but do not report variation in age at morbidity onset. Variation was highlighted by Fries (1980) as crucial for identifying disease postponement. Using incidence of first hospitalization after age 60, as one working example, we estimate variation in morbidity onset over a 27-year period in Denmark. Annual estimates of first hospitalization and the population at risk for 1987 to 2014 were identified using population-based registers. Sex-specific life tables were constructed, and the average age, the threshold age, and the coefficient of variation in age at first hospitalization were calculated. On average, first admissions lasting two or more days shifted towards older ages between 1987 and 2014. The average age at hospitalization increased from 67.8 years (95% CI 67.7–67.9) to 69.5 years (95% CI 69.4–69.6) in men, and 69.1 (95% CI 69.1–69.2) to 70.5 years (95% CI 70.4–70.6) in women. Variation in age at first admission increased slightly as the coefficient of variation increased from 9.1 (95% CI 9.0–9.1) to 9.9% (95% CI 9.8–10.0) among men, and from 10.3% (95% CI 10.2–10.4) to 10.6% (95% CI 10.5–10.6) among women. Our results suggest populations are ageing with better health today than in the past, but experience increasing diversity in healthy ageing. Pensions, social care, and health services will have to adapt to increasingly heterogeneous ageing populations, a phenomenon that average measures of morbidity do not capture.

**Electronic supplementary material:**

The online version of this article (10.1007/s10654-020-00642-3) contains supplementary material, which is available to authorized users.

## Background

Remaining life expectancy at age 60 has increased across developed countries [1]. Whether the extra years of life are spent in good or bad health remains unclear, and depends on how health is measured [2–7]. Fries proposed a scenario where ‘the amount of disability can decrease as morbidity is compressed into the shorter span between the increasing age at onset of disability and death’ [8]. Gruenberg was more pessimistic, arguing that technological advancement would allow people to live for longer but in a prolonged state of poor health [9]. Manton suggested that falling mortality rates would be associated with a change in the distribution of disease types [10]. Specifically, an increase in the number of years spent with moderate health conditions and a decrease in the number of years spent with serious health conditions. The typical approach for evaluating which scenario might be emerging as populations are ageing, is to compare disability-free life expectancy (DFLE) or healthy life expectancy (HLE) with life expectancy [11–14]. If gains in DFLE or HLE are smaller than gains in life expectancy, then morbidity expansion is likely. If gains in DFLE or HLE are greater than gains in life expectancy, morbidity compression is likely.

Key to Fries (1980) theory of morbidity compression is that the years spent with bad health or disability would become increasingly concentrated at the end of life. This implies that the age at morbidity onset should become increasingly homogenous within populations. This dimension is well established in mortality compression research, which focuses on changes to the variation in age at death [15, 16]. However, changes to the variation in age at morbidity onset have largely been overlooked in the morbidity compression debate. This has important implications beyond theory. For individuals, variation in age at morbidity onset represents the amount of uncertainty in the timing of health deterioration. At the macro-level, pensions, social care, and health services will have to adapt to the heterogeneous needs of ageing populations, something that average measures cannot identify [16].

Reducing rates of morbidity onset at any age increases the average age at morbidity onset. Variation in age at onset reductions depend on the balance between reducing rates at younger ages, which compresses the age-at-morbidity-onset distribution into a narrower age span, and reducing rates at older ages, which increases variation by pulling out the right tail of the age-at-morbidity-onset distribution. For variation in the age at morbidity onset to decrease, onset at younger ages needs to be reduced faster than onset at older ages [16]. This would also ensure that the average age at morbidity onset increases.

There is no universal indicator of morbidity. Most estimates of DFLE or HLE use prevalence based estimates of disability or functional limitations, typically derived from self-reported health surveys [11, 17]. In order to estimate variation in the age at morbidity onset, it is necessary to have incidence based estimates of morbidity onset [7]. This requires longitudinal data, as opposed to cross-sectional data, which are rarely available for entire populations. The growth in access to administrative healthcare data, which can be linked with information on the underlying background population, may provide new opportunities for identifying indicators of morbidity beyond the concepts of self-reported disability or functional limitations [6]. Hospital admissions are one potential source of data which are suitable for estimating the incidence rate of medically diagnosed conditions for entire populations [18–21], and have been used to examine temporal changes in population-level health [22–24].

In this paper, we calculate variation in the age at morbidity onset, using hospital admissions data for all individuals aged 60 + between 1987 and 2014 in Denmark, as a working example. The ageing population structure in Denmark is comparable to many other developed countries. Unique to Denmark is that data exist for constructing individual-level hospitalization trajectories for the total population covering a substantial period of time [18, 25].

## Methods and materials

### Data

We linked individual level records, covering the total Danish population, from the National Patient Register (NPR) with data from the Central Population Register (CPR) using the unique personal identification number (CPR-Number). The NPR contains information on all treatments provided in Danish hospitals since 1977 [25]. Reporting of hospital admissions is compulsory, leading to high levels of completeness and reliability. The CPR includes socio-demographic information on the population alive and residing in Denmark since 1968, including sex, place and date of birth, and date of death [26].

### Study population

**Fig. 1:**
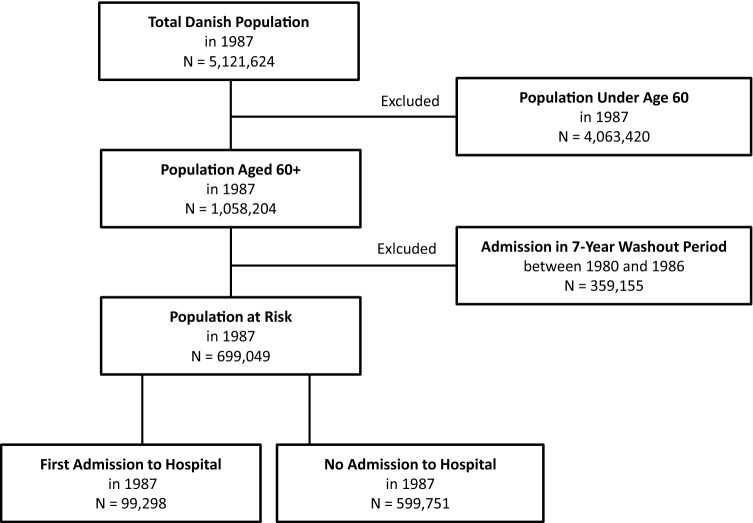
Cohort ﻿study population inclusion and exclusion criteria, using 1987 as an example

As hospitalization data for Denmark began in 1977, we were not able to identify admissions before this year. To ensure comparability throughout the study period, we created an identical cohort study population and consistently applied the same method for each calendar year between 1987 (to allow for the maximum washout period) and 2014. Figure 1 summarizes the process of identifying the study population in 1987 as an example year.

First, we linked CPR and NPR information on all inpatient admissions and the population alive and residing in Denmark aged 60 + . Second, we identified all individuals hospitalized within the previous 7-year period—irrespective of length of stay, and excluded these individuals from the analyses for that particular year. This 7-year washout period was used to limit the chance that a first event is a readmission or a follow-up treatment [20, 27]. Third, from the remaining individuals, we identified the population at risk and the first events within each calendar year. We defined first events as the first inpatient hospitalization after age 60, from all causes, lasting for at least two days. This definition is likely to capture hospital admissions that would require inpatient care consistently throughout the study period. Events were included regardless of whether the outcome was death or discharge. Trends over time in the number of individuals at risk, first events, and those excluded in the washout period are given in Appendix 1 (Supplementary Material).

### Statistical analysis

From the number of first hospital admissions and the population at risk, we estimated age-specific risks of first admission (${q}_{x,t}hosp$), for each age $x$ and each calendar year $t$, for men and women separately. We constructed period life tables for each calendar year using standard demographic methodology [28]. Using age-specific risks to have a first event at age $x$ in year $t$ (${q}_{x,t}hosp$), we estimated ${e}_{x,t}hosp$, which is conditional upon survival to age 60. The definition of ${e}_{x,t}hosp$ is equivalent to the definition of remaining life expectancy at age $x$ in year $t$ (${e}_{x,t}$) in a period life table. It quantifies the remaining average number of years until the event takes place for an individual of exact age $x$, given hospitalization patterns of year $t$. In our case, ${e}_{x,t}hosp$ quantified the expected average number of years until the first hospital admission for a person aged $x$ in year $t$. Adding 60 to the value of ${e}_{x,t}hosp$ allowed the interpretation to be average age at first hospital admission.

Increases in the average age at first hospital admission are achieved when admissions at any age are reduced. Variation in age at first hospital admission can only be reduced when admissions at younger ages are reduced faster than admissions at older ages. Separating younger and older ages is a unique threshold age, which is specific to each distribution and varies over time, but is typically below the average age of admission [29–31]. We calculated the threshold age, by formally identifying the age which a reduction in the age-specific morbidity rate has no impact on variation [32] and report the percentage of admissions below and above the threshold age.

We quantified the amount of variation in age at first hospital admission using the coefficient of variation (CoefV) and reported values as a percentage. The CoefV is a standard measure of dispersion and is defined as the ratio of the standard deviation to the average. Here, the CoefV reflects the variability in age at first hospital admission relative to the average age at first hospital admission. We calculated 95% Confidence Intervals (95% CI) for ${e}_{x,t}hosp$ and CoefV [33].

## Results

### Trends in average age at first admission

**Fig. 2:**
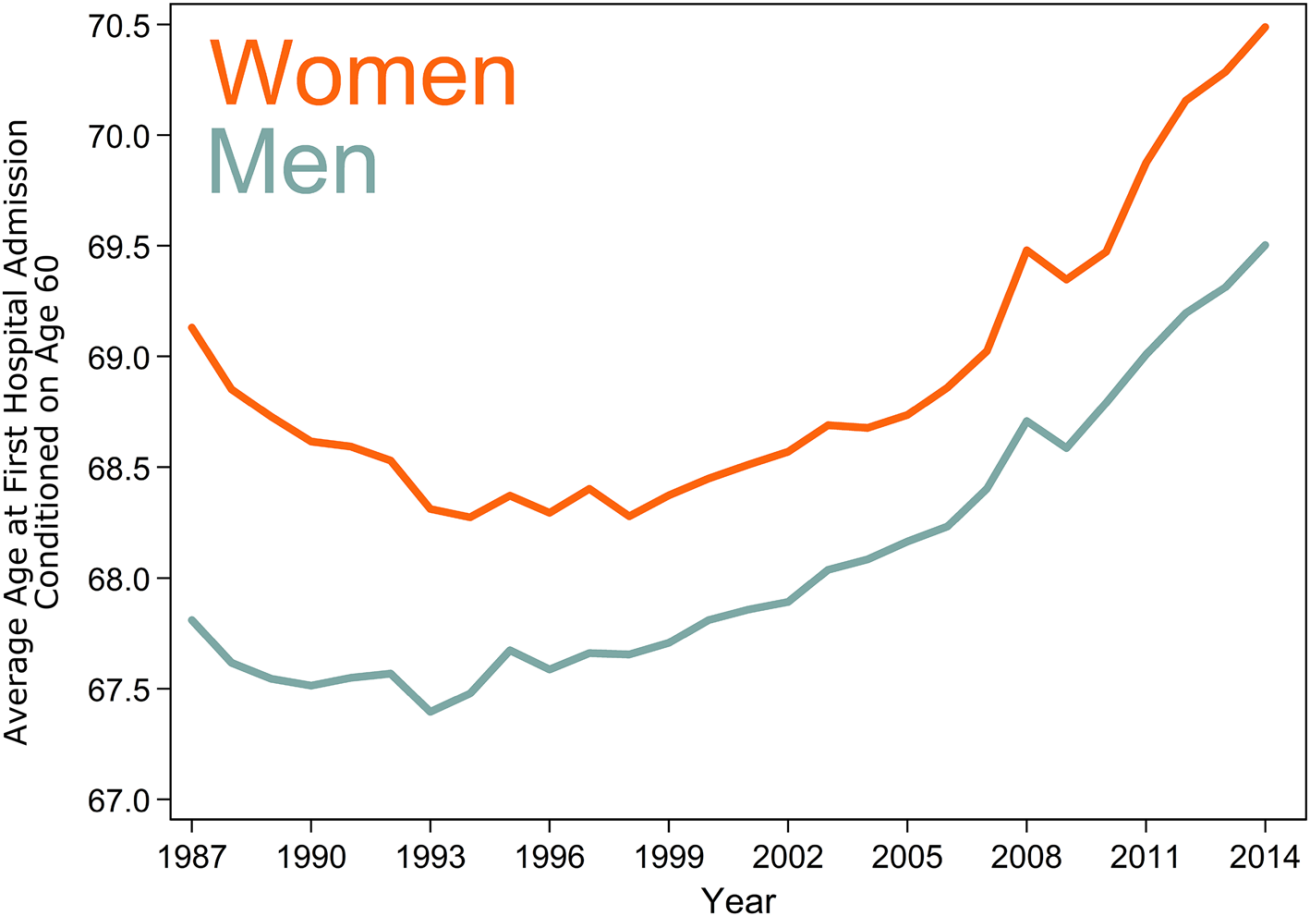
Average age at first hospital admission for men and women between 1987 and 2014

Figure 2 shows trends in the average age at first hospital admission for men and women.

The trend declined throughout the 1990s before rebounding in the 2000s, resulting in a subtle u-shaped pattern. In 1987, the average age at first admission for men was 67.8 years (95% CI 67.7–67.9). At the midpoint, 2001, the average age had increased only slightly to 67.9 years (95% CI 67.8–67.9). By 2014, the average age at first admission had increased to 69.5 years (95% CI 69.4–69.6). For women, the average age decreased slightly between 1987 and 2001: from 69.1 years (95% CI 69.1–69.2) to 68.5 years (95% CI 68.4–68.6). By 2014, the average age for women had increased to 70.5 years (95% CI 70.4–70.6).

### Changes to the age distribution and premature admissions

**Fig. 3:**
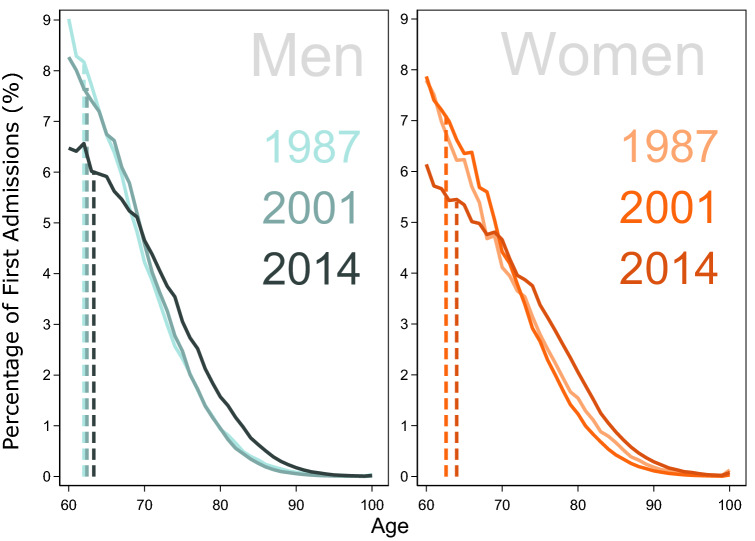
Percentage of first hospital admissions over age and threshold age for men and women in 1987, 2001, and 2014

Figure 3 shows the age distribution of first hospital admissions for men and women in 1987, 2001, and 2014. For each of the three years, the threshold age is plotted as a vertical, dashed line.

The threshold age and the proportion of individuals experiencing an admission below the threshold age increased over time among men and women. In 1987, the threshold age was 62.0 years for men and 17.3% experienced their first admission to hospital below the threshold age. The remaining 82.7% experienced a first admission to hospital above the threshold age. By 2001, the threshold age for men had increased to 62.4 years and the proportion which experienced a first admission to hospital below the threshold age increased to 19.3%. In 2014, the threshold age for men increased further to 63.3 years and 21.3% experienced a first admission to hospital below the threshold age. For women, the threshold age in 1987 was 62.6 years and 19.5% of women experienced a first admission to hospital below the threshold age. The remaining 80.5% of women experienced a first admission to hospital above the threshold age. By 2001, the threshold age for women and the percentage of women being admitted to hospital below the threshold age remained unchanged. The threshold age reached 64.0 years in 2014 and the percentage of women who experienced a first admission to hospital below the threshold age was 22.9%. The remaining 77.1% experienced a first admission to hospital above the threshold age.

### Trends in the coefficient of variation

**Fig. 4:**
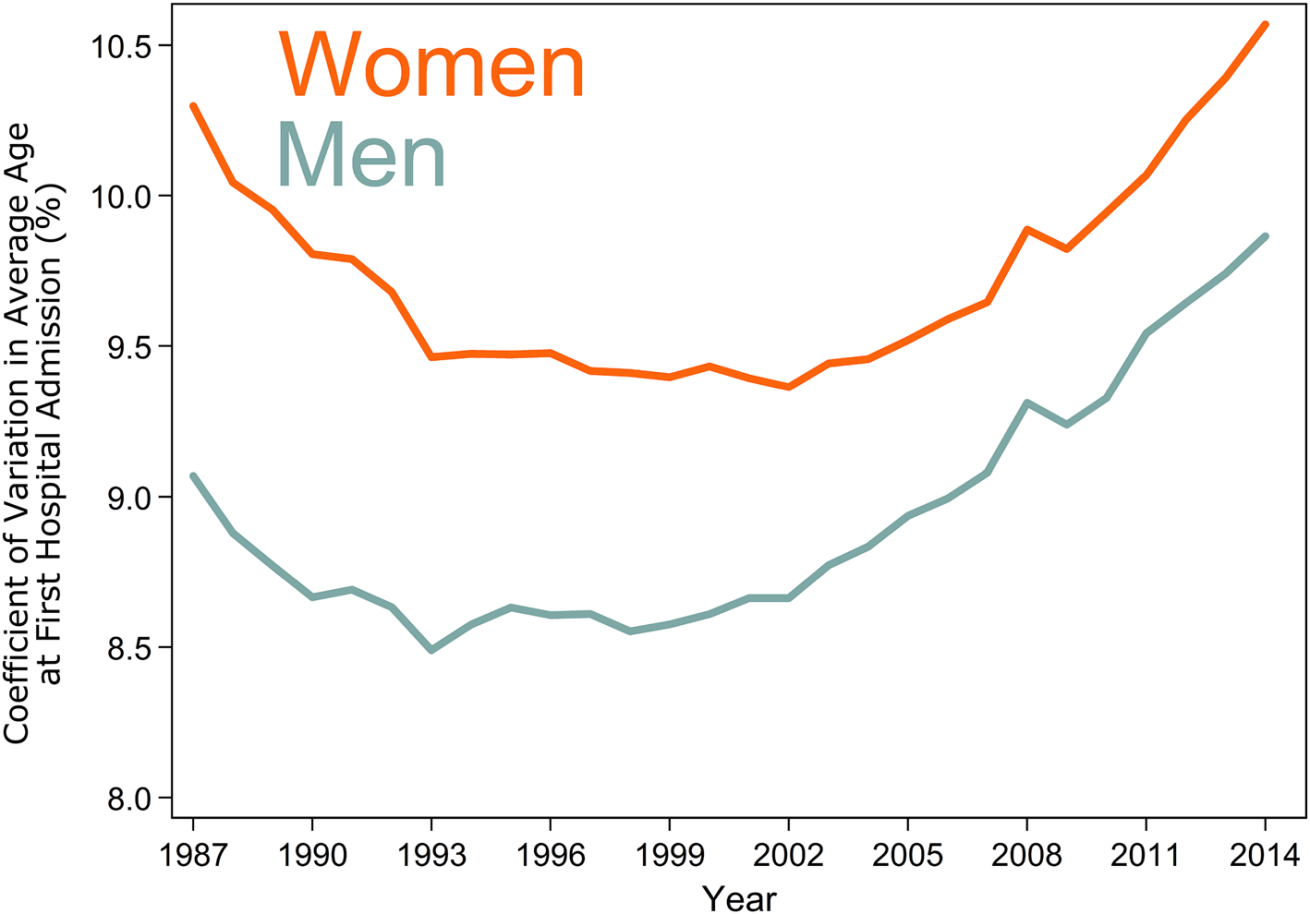
Variation in average age at first hospital admission for men and women between 1987 and 2014

We estimated the CoefV to quantify variation in the average age at first hospital admission. Figure 4 shows trends in the CoefV.

Among men, the CoefV in average age at first admission to hospital decreased from 9.1% (95% CI 9.0–9.1) in 1987 to 8.7% (95% CI 8.6–8.7) in 2001. For women, the corresponding change was from 10.3% (95% CI 10.2–10.4) to 9.4% (95% CI 9.4–9.5). In the early 2000s, the trends in variation changed to slightly increasing. In 2014, the CoefV was 9.9% (95% CI 9.8–10.0) for men and 10.6% (95% CI 10.5–10.6) for women.

### Sensitivity

Our main results reflect hospital stays lasting a minimum of 2 days, including all fatal events. We tested the sensitivity of our results by systematically altering three features of the analysis: (I) varying the minimum length of stay, (II) excluding fatal cases that occurred during admission, and (III) changing the washout period length. The substantive conclusions were generally consistent across the different sensitivity tests. However, the choice of cut-off for length of stay did have an impact. Stays lasting 1 day showed a slightly decreasing trend as opposed to the increasing trend found for all other lengths of stay that were analyzed. Results of sensitivity analyses are provided in Appendices 2 and 3 (Supplementary Material).

## Discussion

### Summary of findings

We found the average age at first hospital admission at ages 60, for admissions lasting more than 2 days, to be higher in 2014 than in 1987, suggesting that people on average tend to live for longer without being admitted to hospital. At the same time, there may have been an increase in the proportion of first events occurring below the threshold age, and variation in age at first hospital admission may have increased. These findings indicate that individuals may face greater uncertainty in the timing of morbidity onset and that health at older ages may have become increasingly heterogeneous.

### Interpretations and implications

A shift of hospital admissions towards older ages would suggest that people are ageing with better health today than in the past. Healthy ageing is due to multiple factors, including improved health during childhood, reduced exposure to hazardous working conditions, and changes in health behaviors such as smoking or diet [34, 35]. Another contributing factor is technological advancement which has enabled more individuals to survive longer in better health. Although some chronic diseases may show increased prevalence over time, they may now lead to a hospital admission later in life. Perhaps the strongest example of this is the treatment of cardiovascular diseases [36, 37]. Treatment has changed dramatically over time and mortality has declined, leading to more people surviving without serious cardiovascular events and only being admitted to hospital later in life.

At the same time, increasing variation in age at morbidity onset would indicate increasing diversity in healthy aging. The same technological advancements that have postponed adverse health events, have also enabled individuals to live for longer while managing chronic conditions, resulting in populations with more heterogeneous health profiles [9, 38, 39]. Additionally, some component of increasing health variation could come from changes to the epidemiologic environment experienced by older adults as infants and children. As control over infectious diseases has improved, weaker individuals, who would have died as children in previous decades, have survived to older ages, making for a more heterogeneous population [39].

Changes to population health are not the only factor which could have contributed to the changes in hospital admissions. Differences over time in admission and treatment strategies will have had an impact [24]. In an attempt to identify changes in admission and treatment strategies, we examined 19 causes of admission using harmonized ICD-8 and ICD-10 codes [Appendix 4 (Supplementary material)]. While the relative contribution from some causes decreased over the study period, other causes increased or remained stable. From these data, we were unable to identify any clear changes in admission or treatment strategies. Systematic changes are only likely to be identifiable when looking at diagnostic coding in even finer detail than was available. However, increasing the level of diagnostic detail would risk increasing potential misclassification bias [40]. An alternative approach would have been to compare detailed information on inpatient, outpatient, and primary healthcare attendances by cause of admission and length of treatment. Unfortunately, in Denmark, outpatient data and primary healthcare data do not contain suitable information for carrying out such analyses.

### Conceptual and methodological considerations

Studies of morbidity traditionally use self-reports of functional limitation, disability, or poor health from survey data to capture the concept of morbidity [4, 6, 41–44]. Empirical measures have diversified to encompass a range of dimensions including; level of functioning (impairment, functional limitation or activity restriction), severity of disability (severe or mild) and type of impairment (physical or cognitive) [44]. Self-reported measures of health and disability give individuals the opportunity to share more information about their own health than objective measures capture. At the same time, self-reported measures of health and disability may be vulnerable to recall bias and systematic differences in reporting behaviors [45–47]. While there is no consensus surrounding the most appropriate indicator of morbidity, a common feature is the intention to capture the impact disease has on daily life. However, recommendations have been made to (i) develop the concept of morbidity to better account for chronic conditions that do not result in functional limitation or disability and (ii) identify alternative data for capturing morbidity onset [6]. We have further highlighted that variation in age at onset, a key concept for monitoring mortality compression [15, 16, 48], has largely been overlooked in the morbidity compression debate.

Unfortunately, there are few longitudinal data sources covering an entire population that enable the identification of morbidity onset required to estimate variation. Even for a country with an established register based system, such as Denmark, options were limited. We identified first admission to hospital after age 60 as one illustrative example. From hospital admissions data we were able to estimate the incidence rate of a medical diagnosis as a proxy indicator of morbidity [18, 19]. However, the administrative data used in this study were not free from limitations.

In Denmark, General Practitioners are gate keepers for determining access to hospital care [25, 49] and hospitals are not the only setting where a medical diagnosis can occur. Many chronic conditions, which develop slowly, may be diagnosed in primary healthcare or outpatient settings and our study will have underestimated the full burden of morbidity. A restriction of our methodological approach, is that it is not directly applicable to international sources of administrative healthcare data that only capture individuals from the point of engagement [50]. A final limitation of this study is that changing the length of stay impacted the conclusions of the paper [Appendix 2 (Supplementary Material)]. Our main results defined an admission as an inpatient stay lasting for a minimum of 2 days. We found a stepwise increase in the average age and variation in age at first admission with every increase in minimum length of stay. Hospital stays lasting 1 day were an exception and showed a slightly decreasing trend. Therefore, measuring variation in age at morbidity onset from alternative administrative data is necessary. Medical diagnosis estimates from prescriptions data or primary healthcare data may yield very different results. The secular trends for increasing diagnostic activity and lower thresholds for hypertension and cholesterol may have decreased the age of morbidity onset and variation in age at onset in relation to primary healthcare diagnosis. At the same time, it is likely that these prevention effects may have contributed to the increase in age at morbidity onset and variation we identified using hospitalization data. Where possible, we encourage variation in age at morbidity onset to be routinely measured alongside average indicators of morbidity onset. Considering that variation in age at death has increased at older ages, increasing population level heterogeneity in age at morbidity onset is not an unlikely scenario [15, 39].

### Concluding theoretical reflections

Morbidity compression studies have consistently calculated the average number of years spent in an unhealthy state, compared with life expectancy [5, 51–55]. However, Fries argued that the ‘analysis of variation, not of average values, becomes crucial’ [8] for understanding disease postponement. Although it has been forty years since Fries made this observation, empirical measurement of variation in morbidity onset has been overlooked. Incorporating variation in age at morbidity onset is important for individual life planning and population-level welfare: Pensions, social care and health services will have to adapt to the heterogeneous needs of ageing populations, something that average morbidity measures cannot identify.

## Electronic supplementary material

Below is the link to the electronic supplementary material.

Supplementary file1 (PDF 253 kb)

Supplementary file2 (PDF 1744 kb)

Supplementary file3 (PDF 749 kb)

Supplementary file4 (PDF 273 kb)
