# Supplementary figures and images for: Rethinking morbidity compression
Source: Eur J Epidemiol. 2020 May 16;35(5):381–8. doi: 10.1007/s10654-020-00642-3 (PMC7250949; doi:10.1007/s10654-020-00642-3)

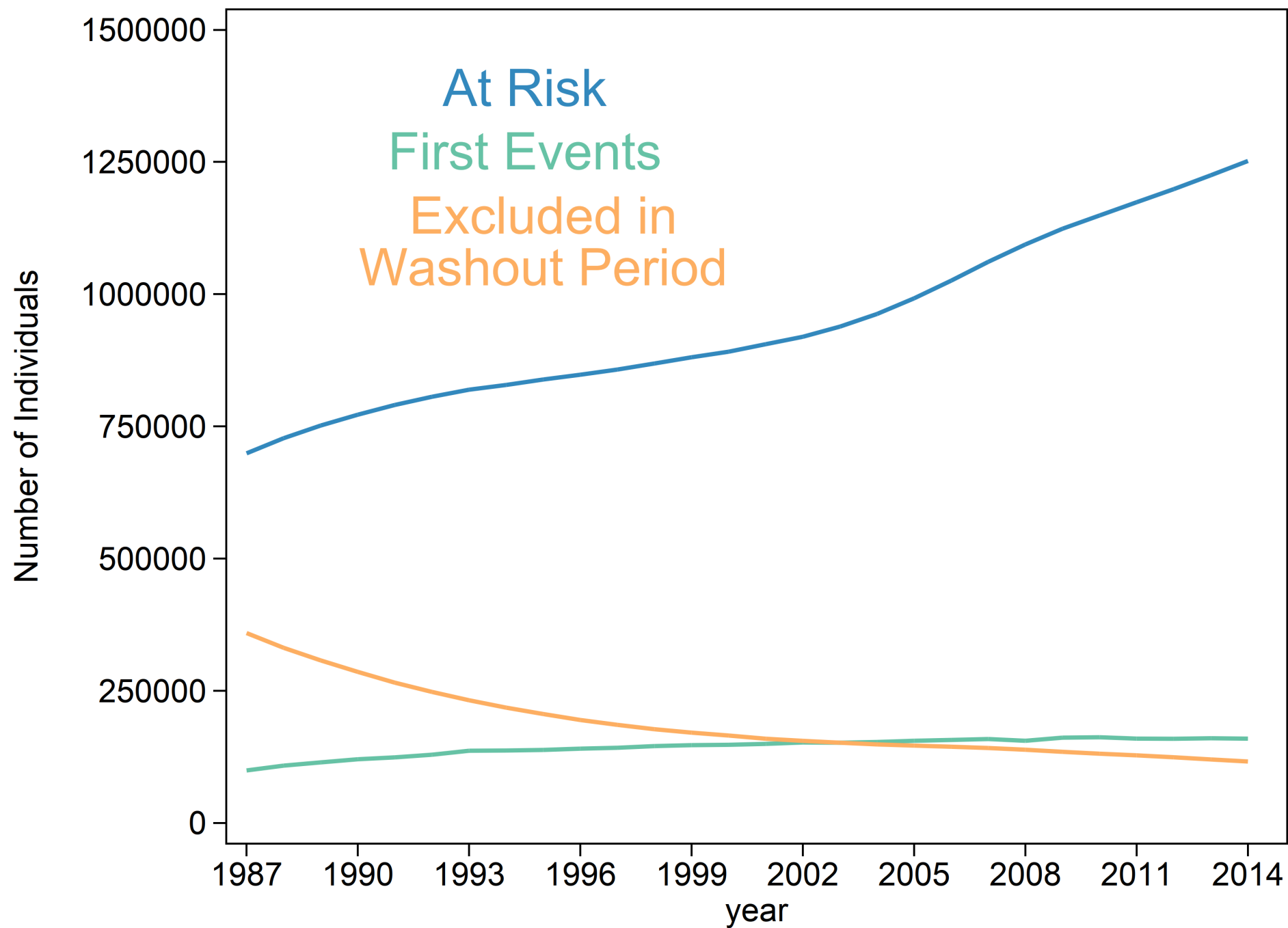

Supplement: Supplementary file 1 — Supplementary file1 (PDF 253 kb) [file 10654_2020_642_MOESM1_ESM.pdf]

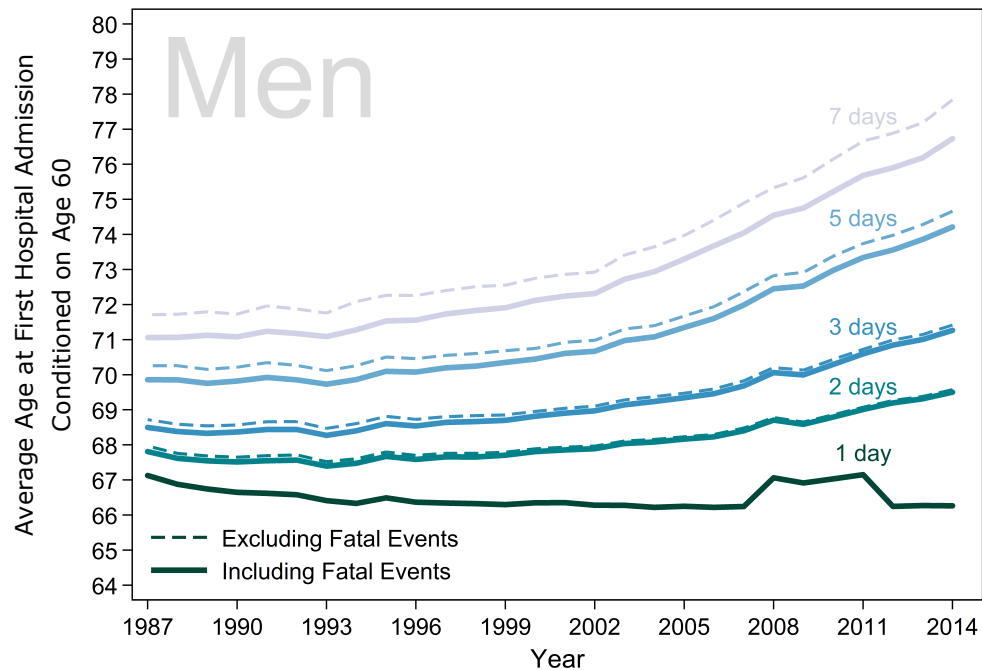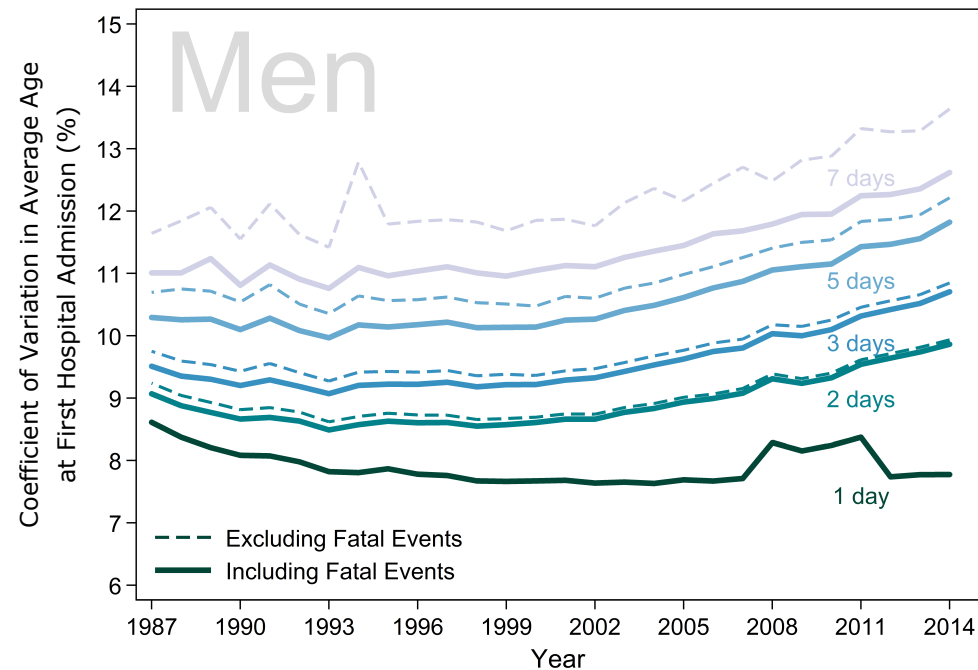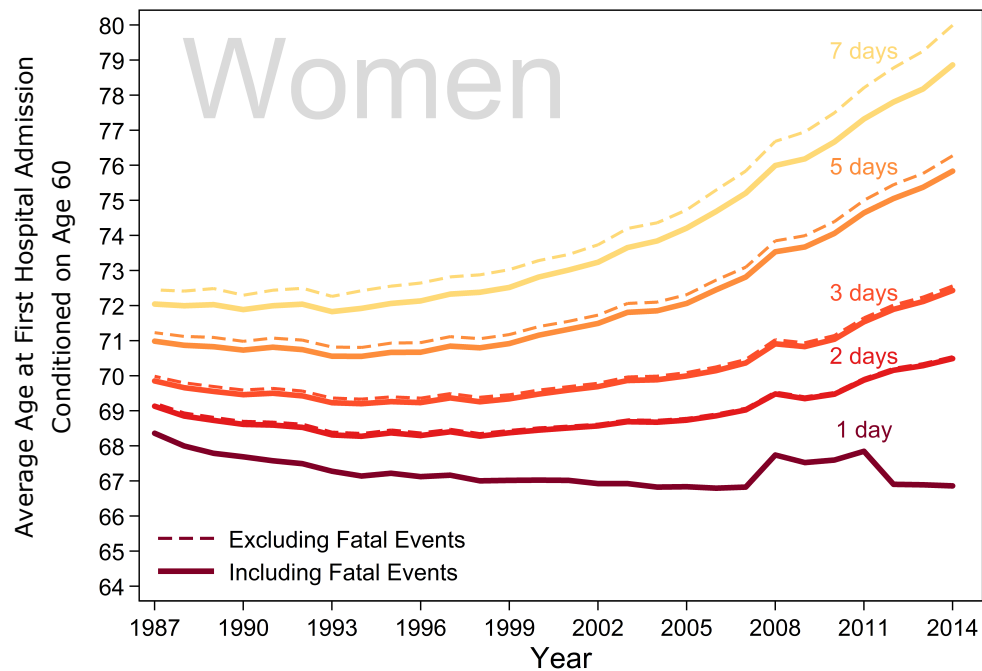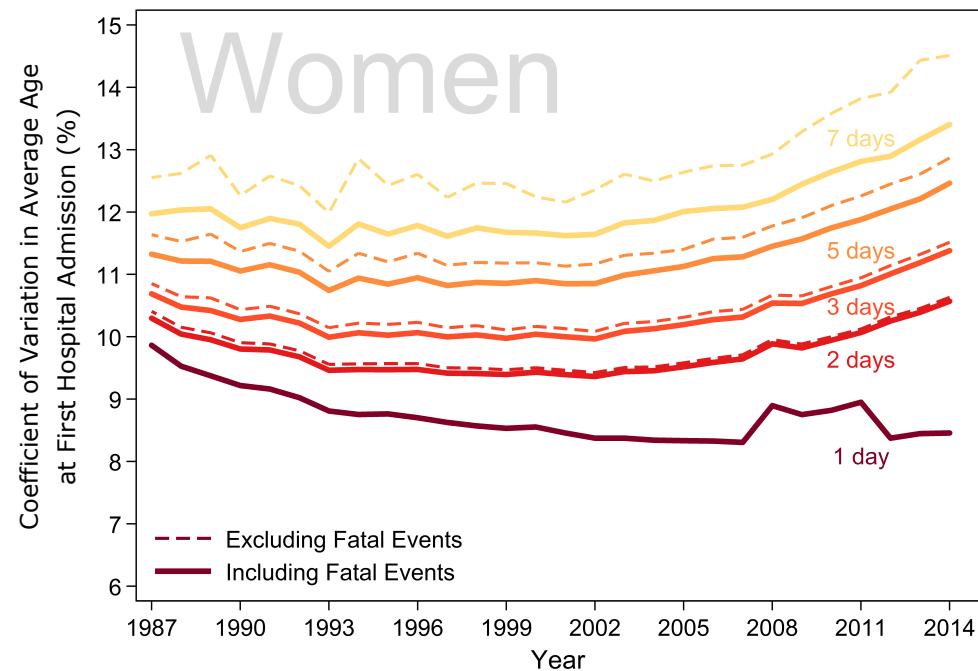

Supplement: Supplementary file 2 — Supplementary file2 (PDF 1744 kb) [file 10654_2020_642_MOESM2_ESM.pdf]

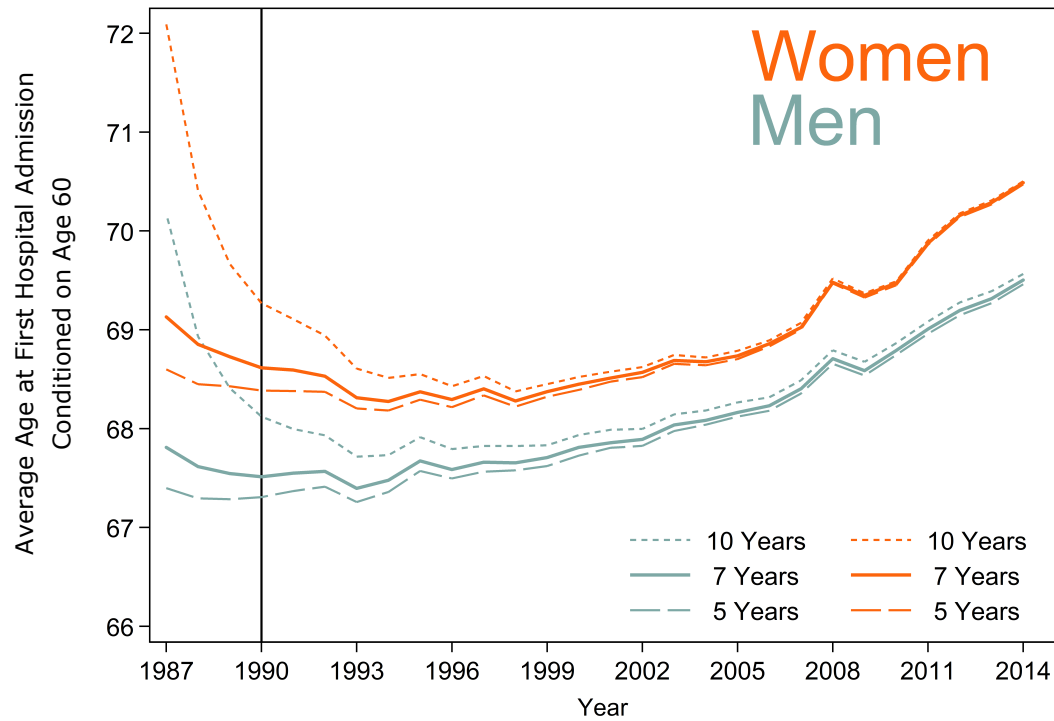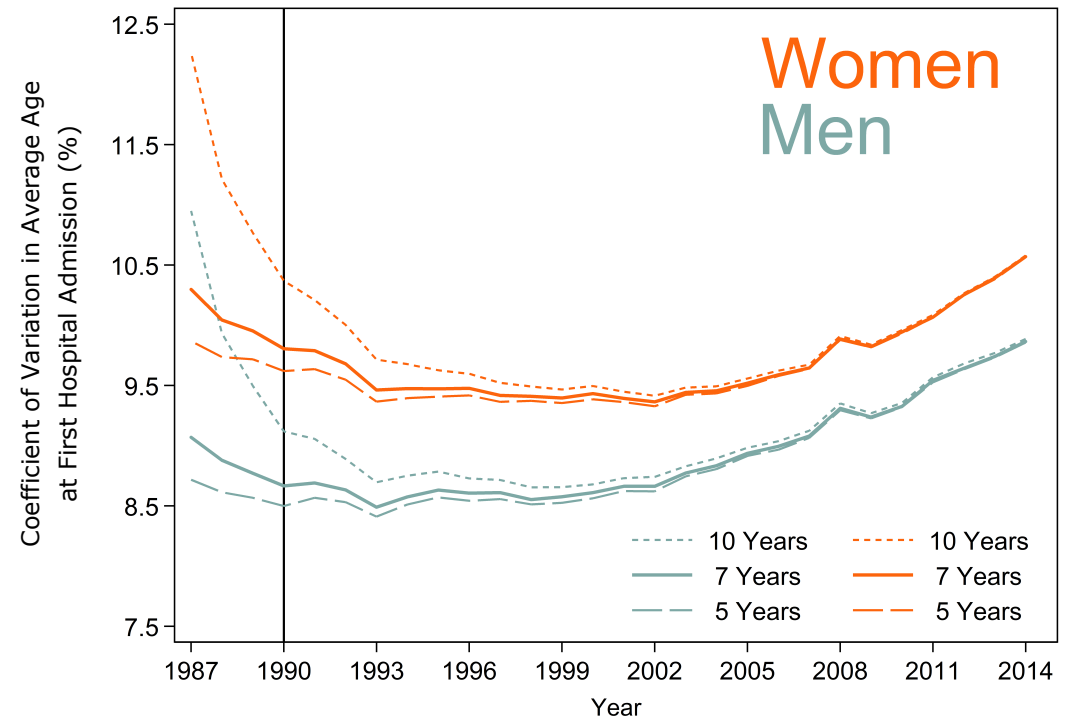

Supplement: Supplementary file 3 — Supplementary file3 (PDF 749 kb) [file 10654_2020_642_MOESM3_ESM.pdf]

Percentage of First Admissions

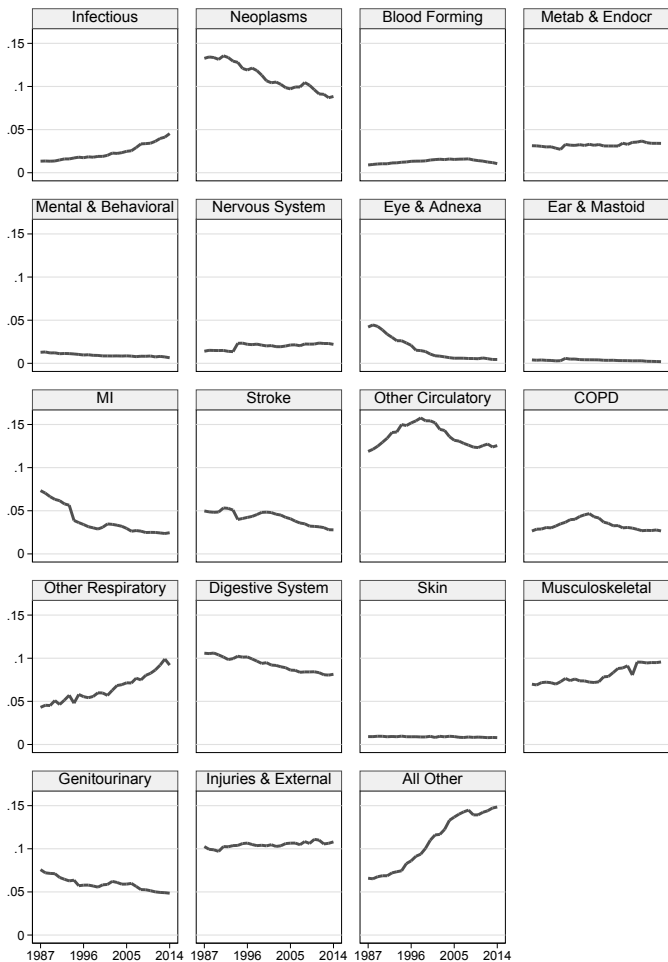

Year

Supplement: Supplementary file 4 — Supplementary file4 (PDF 273 kb) [file 10654_2020_642_MOESM4_ESM.pdf]
